# Clinical characterization of endometriosis phenotypes

**DOI:** 10.1007/s00404-025-08191-4

**Published:** 2025-10-17

**Authors:** Louisa Hofbeck, Katharina Au, Simon Blum, Nadezda Sipulina, Laura Lotz, Johannes H. Lermann, Stefan P. Renner, Peter A. Fasching, Matthias W. Beckmann, Stefanie Burghaus

**Affiliations:** 1 Department of Gynecology and Obstetrics, Erlangen University Hospital, Universitäts-Endometriosezentrum Franken, Friedrich-Alexander-Universität Erlangen-Nürnberg, Universitätsstr. 21-23, 91054 Erlangen, Germany; 2 Department of Gynecology and Obstetrics, Hospital Klagenfurt, Klagenfurt, Austria; 3 Department of Gynecology and Obstetrics, Boeblingen Clinic, Hospital Sindelfingen-Böblingen, Böblingen, Germany

**Keywords:** Endometriosis, Adenomyosis, Symptoms, Pelvic pain, Dyschezia, Classification

## Abstract

**Purpose:**

Endometriosis appears in various forms and symptoms. With regard to the established endometriosis classifications, it is hardly possible to draw conclusions from the endometriosis to the symptoms caused by it. The objective of this study was to evaluate whether different endometriosis phenotypes are associated with distinct pain profiles.

**Materials and methods:**

3329 patients underwent surgical treatment for endometriosis at the University Endometriosis Center Franconia of the Erlangen University between September 2011 and January 2024. They were grouped by phenotype [superficial (SE), deep (DIE) and adenomyosis (AM)] and assessed for pelvic pain, dyspareunia, dysuria and dyschezia. The study examined pain distribution across phenotypes and pain intensity among symptomatic patients (NRS > 0).

**Results:**

Patients with SE only reported pelvic pain less frequently and with lower intensity than those with additional AM Groups SE/AM and SE/DIE/AM. Dyspareunia was less common in Group SE only vs. Group SE/AM; pain intensity was highest in AM only and lowest in Group SE/DIE. Dysuria was most frequent in Group SE/DIE/AM, with no significant intensity differences. Dyschezia was more frequent in Group SE/DIE/AM and less in Group SE only, again without significant intensity differences.

**Conclusion:**

Pain frequency and intensity differed by endometriosis phenotype. SE showed the lowest pain frequency and pelvic pain intensity. AM, especially with other subtypes, was linked to higher frequency and intensity of pelvic pain, as well as more dyspareunia and dysuria. DIE was mainly associated with more frequent dyschezia, but not with increased pelvic pain intensity.

**Supplementary Information:**

The online version contains supplementary material available at 10.1007/s00404-025-08191-4.

## Introduction

Endometriosis is a heterogeneous disease, typically presenting with symptoms such as dysmenorrhea, dyschezia, dysuria, dyspareunia, chronic pelvic pain and infertility [1]. The diagnosis of endometriosis by visual diagnosis during laparoscopy or histological confirmation is still widespread. In the current ESHRE guideline from 2022, a diagnosis by view is even used over histology. However, laparoscopy is only recommended in cases where it was not possible to diagnose endometriosis using imaging [2]. Adenomyosis can be diagnosed using a combination of clinical assessment and imaging techniques (transvaginal ultrasound as first-line modality or MRI) or histologically [3, 4]. The prevalence of endometriosis is not sufficiently known, as the diagnosis is made by surgery or by imaging and non-symptomatic women are not examined [5]. Nevertheless, a prevalence of approx. 10% can be assumed [6].

Endometriosis has many manifestations and is described using various classifications. Widely used are rASRM [7], EFI [8] and #Enzian [9, 10]. The rASRM score provides a rough overview of the extend of the lesions. However, these scores, especially the rASRM, cannot be used to derive any symptoms, disease progression or predictions [11–17]. Some data suggest a more severe impairment in quality of life for higher rARSM stages [18]. The #Enzian classification obtained in non-invasive diagnostics has a high correlation with the surgically determined classification and can therefore be used for individualized treatment planning. However, there is not yet enough data available to evaluate the #Enzian in terms of symptom correlation [19].

A phenotype-based classification could provide a more comprehensive approach by integrating key factors such as lesion morphology. In the future, there is a need to move away from artificial classifications to distinguish different subgroups towards the formation of subgroups based on clinical characteristics. This could as well include factors such as inflammatory profile, pain patterns, hormonal responses, psychological disorders and molecular signatures [20]. This would allow for more individualized treatment strategies, better prediction of disease progression, recurrence rates and better stratification in clinical trials.

In this study, we compare patients with different types of endometriosis and examine them with regard to their symptoms. This should contribute to a better understanding of endometriosis and enable a more individualized therapy.

## Methods

As part of the International Endometriosis Evaluation Program (IEEP) [21], which is designed as a prospective cohort study, all patients presenting for surgical therapy due to suspected endometriosis or for surgical treatment of a previously diagnosed endometriosis at the University Endometriosis Center Franconia of the Erlangen University are recorded. All patients were given a structured pre-operative questionnaire on their medical history, symptoms, previous surgical and drug therapies, as well as questions on fertility and reproduction. This information was supplemented postoperatively by clinical data from the patient file. Main symptoms include infertility or pain symptoms or both or suspicious results. Data are collected using a web-based remote data entry system (electronic case report form, eCRF) based on the patient file and a structured preoperative questionnaire. The data were transferred to an MS Access database for further analysis.

For this prospective case-only study, patients between September 2011 and February 2024 were evaluated. We designed the study as a case-case analysis with a total of 7 groups. We categorized the patients based on their clinical presentation [22]. Each patient could have superficial endometriosis (SE), deep infiltrating endometriosis (DIE) or adenomyosis (AM) alone or in combination. Ovarian endometriosis was classified as SE, as it typically does not exhibit infiltration > 5 mm.

**Fig. 1:**
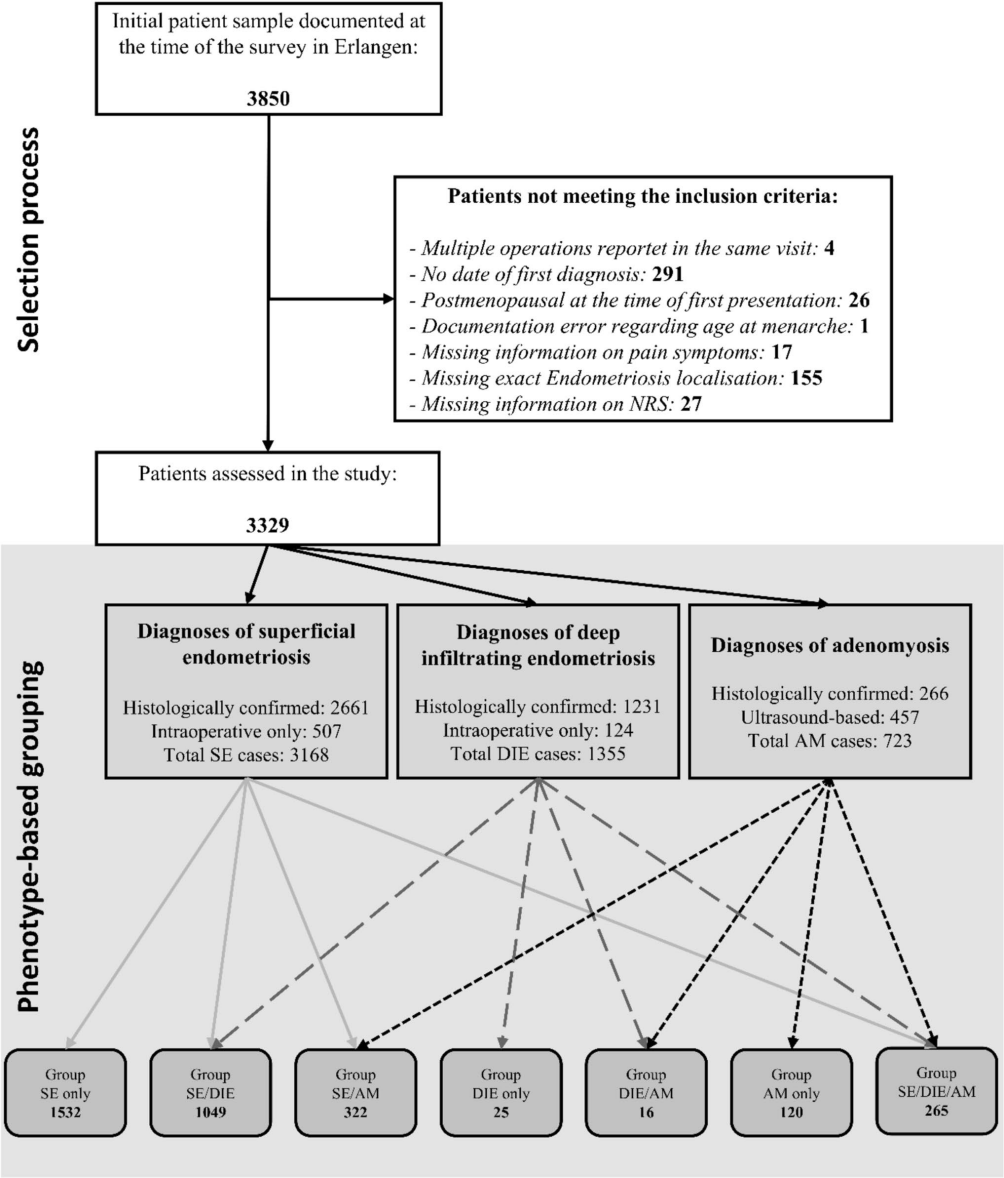
PRISMA flow chart of patient selection and phenotype-based grouping. SE: superficial endometriosis, DIE deep infiltrating endometriosis. AM: adenomyosis

In our study, we examined the presence of phenotypes, but not their extent or classification. Given the absence of deep tissue infiltration and the histological similarity to SE, ovarian endometriosis was considered within the context of SE. In accordance with current guidelines, SE and DIE were diagnosed intraoperatively and did not necessarily require confirmatory histology in general [23]. AM, on the other hand, was diagnosed either clinically using imaging techniques or histologically by hysterectomy. The imaging procedure of choice was structured TVUS in a specialized ultrasound department of the Women’s Hospital. For the sonographic assessment of AM, the MUSA criteria [24, 25] for the sonographic diagnosis of AM were used and supplemented by the “question-mark sign” [3]. For the diagnosis of AM, at least one of the above criteria had to be met. Based on the combinations of the three phenotypes (SE, DIE and AM), patients were classified into seven mutually exclusive groups (SE only; SE/DIE; SE/AM; DIE only; DIE/AM; AM only; SE/DIE/AM). Among the 3168 patients diagnosed with SE, 2661 cases were confirmed through intraoperative inspection and subsequent histological verification, while 507 were diagnosed only intraoperatively. In the case of DIE, 1231 diagnoses were supported by histology in addition to intraoperative findings, and 124 were based on intraoperative diagnosis alone. AM was diagnosed only by transvaginal ultrasound in 457 patients and in 266 cases by histology (Fig. 1).

All patients included in the study were asked on the basis of a preoperative questionnaire whether they had suffered from complaints such as “lower pelvic pain/dyspareunia”, “dyspareunia profunda”, “dysuria” or “dyschezia” in the last 3 months. If this was the case, patients were asked to quantify this pain using an NRS [26]. An NRS of 0 (= no complaints) was assumed for patients who reported no symptoms overall or no pain for the corresponding pain type. For pelvic pain / dyspareunia, patients indicated the timing of symptoms in relation to menstruation. To increase group size, data on pelvic pain and dysmenorrhea were analyzed jointly.

### Patient selection

3850 patients were available for the evaluation. Four patients were excluded because more than one surgery was reported in the same visit. No adequate date of first diagnosis could be calculated for 291 patients. 269 patients were not premenopausal at first presentation. In one patient, there was a documentation error regarding age at menarche. 17 patients were excluded due to missing information on pain symptoms. A further 155 patients were excluded because the type of endometriosis could not be clearly assigned to a group consisting of SE, DIE and AM. 27 patients were excluded if information was available on whether the patient had experienced symptoms in the last 3 months, but no information was available on the intensity of the pain. After applying all exclusion criteria, 3329 patients were included in the study (Fig. 1).

### Statistical considerations

This study aimed to assess whether endometriosis phenotypes are associated with distinct pain profiles. The frequencies of pelvic pain, dyspareunia, dysuria, and dyschezia were compared across phenotypes using chi-square tests. Pain was recorded as a categorical variable (yes/no); responses of “unknown” were excluded. There was considerable variation in group sizes. Table 1 summarizes pain frequencies by group. A two-sided *p* < 0.05 was considered significant. For significant results, adjusted standardized residuals (*z* >|2.93| after Bonferroni correction) identified contributing cells. Pairwise chi-square tests with Bonferroni-adjusted significance levels were then performed.

**Table 1:** Frequency of pain symptoms

	Group SE only (*n* = 1532)	Group SE/DIE (*n* = 1049)	Group SE/AM (*n* = 322)	Group DIE only (*n* = 25)	Group DIE/AM (*n* = 16)	Group AM only (*n* = 120)	Group SE/DIE/AM (*n* = 265)
Pelvic pain							
Yes	*n* = 1218 (79.5%)	*n* = 892 (85.0%)	*n* = 301 (93.5%)	*n* = 18 (72.0%)	*n* = 14 (87.5%)	*n* = 107 (89.2%)	*n* = 247 (93.2%)
No	*n* = 314 (20.5%)	*n* = 156 (14.9%)	*n* = 21 (6.5%)	*n* = 7 (28.0%)	*n* = 2 (12.5%)	*n* = 13 (10.8%)	*n* = 18 (6.8%)
Unknown	*n* = 0 (0.0%)	*n* = 1 (0.1%)	*n* = 0 (0.0%)	*n* = 0 (0.0%)	*n* = 0 (0.0%)	*n* = 0 (0.0%)	*n* = 0 (0.0%)
Dyspareunia							
Yes	*n* = 637 (41.6%)	*n* = 468 (44.6%)	*n* = 169 (52.5%)	*n* = 8 (32.0%)	*n* = 7 (43.8%)	*n* = 58 (48.3%)	*n* = 134 (50.6%)
No	*n* = 868 (56.7%)	*n* = 569 (54.2%)	*n* = 150 (46.6%)	*n* = 16 (64.0%)	*n* = 8 (50.0%)	*n* = 60 (50.0%)	*n* = 124 (46.8%)
Unknown	*n* = 27 (1.8%)	*n* = 12 (1.1%)	*n* = 3 (0.9%)	*n* = 1 (4.0%)	*n* = 1 (6.3%)	*n* = 2 (1.7%)	*n* = 7 (2.6%)
Dysuria							
Yes	*n* = 233 (15.2%)	*n* = 175 (16.7%)	*n* = 73 (22.7%)	*n* = 4 (16.0%)	*n* = 4 (25.0%)	*n* = 20 (16.7%)	*n* = 63 (23.8%)
No	*n* = 1290 (84.2%)	*n* = 870 (82.9%)	*n* = 247 (76.7%)	*n* = 21 (84.0%)	*n* = 12 (75.0%)	*n* = 99 (82.5%)	*n* = 201 (75.8%)
Unknown	*n* = 9 (0.6%)	*n* = 4 (0.4%)	*n* = 2 (0.6%)	*n* = 0 (0.0%)	*n* = 0 (0.0%)	*n* = 1 (0.8%)	*n* = 1 (0.4%)
Dyschezia							
Yes	*n* = 330 (21.5%)	*n* = 304 (29.0%)	*n* = 92 (28.6%)	*n* = 9 (36.0%)	*n* = 7 (43.8%)	*n* = 31 (25.8%)	*n* = 102 (38.5%)
No	*n* = 1191 (77.7%)	*n* = 740 (70.5%)	*n* = 229 (71.1%)	*n* = 15 (60.0%)	*n* = 9 (56.3%)	*n* = 88 (73.3%)	*n* = 162 (61.1%)
Unknown	*n* = 11 (0.7%)	*n* = 5 (0.5%)	*n* = 1 (0.3%)	*n* = 1 (4.0%)	*n* = 0 (0.0%)	*n* = 1 (0.8%)	*n* = 1 (0.4%)

*SE* superficial endometriosis, *DIE* deep infiltrating endometriosis, *AM* adenomyosis

The study also assessed differences in pain intensity (NRS) across endometriosis phenotypes. Only patients with NRS > 0 were included. Due to non-normal data and small group sizes, the Kruskal–Wallis test was used. Significant results (*p* < 0.05) were followed by pairwise Mann–Whitney *U* tests with Bonferroni correction. Pain intensity was visualized with violin and boxplots; median and mean values were reported. Normality was assessed using the Shapiro–Wilk test and visual inspection. Analyses were performed with SPSS 29.0, R 4.4.3, and RStudio 2024.12.1. Missing values were excluded without imputation.

## Results

### Patient characteristics

All 3329 patients were divided into the seven groups. The groups were then examined with regard to patient characteristics. Due to the clinical setting, there was a difference in group size and in some patient characteristics. The majority of patients had SE with or without concomitant DIE. Noticeable fewer patients had DIE alone or AM. The mean age varied between the groups. Group DIE/AM was 39.1 (SD ± 8.0) years old at first presentation. In contrast, group SE only was 32.3 (SD ± 6.8) years old. In general, the groups with AM presented at an older age than patients without AM. The differences between the groups were also evident in the reason for presentation. Patients in group SE only with 40.7%, group SE/DIE with 46.8% and group SE/DIE/AM with 40.4% showed notably more infertility than other groups. A more uniform picture emerged for the reason for reporting pain, ranging from 78.3% in group SE only to 91.7% in group SE/DIE/AM. Overall, pain was reported very frequently as a reason for presentation. Age at first diagnosis and age at menarche showed also a much more homogeneous picture. Depending on the group, the BMI ranged from 23.8 (SD ± 4.7) to 27.5 (SD ± 6.1) and corresponds to the expected value. The number of pregnancies ranged from 0.47 (SD ± 0.88) in group SE/DIE to 1.19 (SD ± 1.30) in group AM only and correlated well with the number of live births 0.30 (SD ± 0.64) and 0.92 (SD ± 1.10) in the respective groups. Patients in group DIE/AM had by far the most previous operations 1.13 (SD ± 1.41) and group AM only had the most pre-therapies (30.0%). Group SE only with 14.2%, and group DIE only with 12.0% were the least likely to have had prior therapy. Patient characteristics are shown in Table 2.

**Table 2:** Patient characteristics

Patient characteristics	Group SE only (*n* = 1532)	Group SE/DIE (*n* = 1049)	Group SE/AM (*n* = 322)	Group DIE only (*n* = 25)	Group DIE/AM (*n* = 16)	Group AM only (*n* = 120)	Group SE/DIE/AM (*n* = 265)
Reason for presentation							
Pain	*n* = 1199 (78.3%)	*n* = 873 (83.2%)	*n* = 293 (91.0%)	*n* = 20 (80.0%)	*n* = 14 (87.5%)	*n* = 107 (89.2%)	*n* = 243 (91.7%)
Infertility	*n* = 624 (40.7%)	*n* = 491 (46.8%)	*n* = 94 (29.2%)	*n* = 8 (32.0%)	*n* = 5 (31.25%)	*n* = 24 (20.0%)	*n* = 107 (40.4%)
Other	*n* = 368 (24.0%)	*n* = 211 (20.1%)	*n* = 41 (12.7%)	*n* = 5 (20.0%)	*n* = 3 (18.75%)	*n* = 18 (15.0%)	*n* = 34 (12.8%)
Age at first presentation	32.3 (SD ± 6.8)	33.0 (SD ± 6.4)	34.4 (SD ± 8.2)	36.1 (SD ± 5.8)	39.1 (SD ± 8.0)	36.2 (SD ± 8.2)	34.5 (SD ± 7.0)
Age at diagnosis	31.5 (SD ± 6.9)	32.1 (SD ± 6.5)	33.3 (SD ± 8.4)	35.4 (SD ± 5.9)	35.9 (SD ± 9.0)	34.4 (SD ± 8.8)	32.2 (SD ± 7.3)
Age at menarche	12.9 (SD ± 1.5)	12.9 (SD ± 1.5)	13.0 (SD ± 1.5)	12.9 (SD ± 1.5)	13.2 (SD ± 1.4)	12.8 (SD ± 1.6)	12.9 (SD ± 1.5)
BMI	23.8 (SD ± 4.7)	23.9 (SD ± 4.7)	24.4 (SD ± 4.7)	27.5 (SD ± 6.1)	24.8 (SD ± 4.5)	25.4 (SD ± 6.0)	24.3 (SD ± 5.4)
Pregnancies	0.56 (SD ± 0.97)	0.47 (SD ± 0.88)	0.80 (SD ± 1.15)	0.96 (SD ± 1.08)	0.81 (SD ± 0.91)	1.19 (SD ± 1.30)	0.67 (SD ± 0.95)
Life births	0.34 (SD ± 0.71)	0.30 (SD ± 0.64)	0.54 (SD ± 0.88)	0.79 (SD ± 0.93)	0.63 (SD ± 0.81)	0.92 (SD ± 1.10)	0.45 (SD ± 0.78)
Previous operations	0.21 (SD ± 0.56)	0.29 (SD ± 0.63)	0.27 (SD ± 0.74)	0.28 (SD ± 0.61)	1.13 (SD ± 1.41)	0.42 (SD ± 0.75)	0.51 (SD ± 0.82)
Pain at first presentation							
Yes	*n* = 1250 (81.6%)	*n* = 911 (86.8%)	*n* = 308 (95.7%)	*n* = 21 (84.0%)	*n* = 14 (87.5%)	*n* = 109 (90.8%)	*n* = 250 (94.3%)
No	*n* = 282 (18.4%)	*n* = 138 (13.2%)	*n* = 14 (4.3%)	*n* = 4 (16.0%)	*n* = 2 (12.5%)	*n* = 11 (9.2%)	*n* = 15 (5.7%)
Unknown	*n* = 0 (0.0%)	*n* = 0 (0.0%)	*n* = 0 (0.0%)	*n* = 0 (0.0%)	*n* = 0 (0.0%)	*n* = 0 (0.0%)	*n* = 0 (0.0%)
Previous therapy							
Yes	*n* = 217 (14.2%)	*n* = 172 (16.4%)	*n* = 58 (18.0%)	*n* = 3 (12.0%)	*n* = 4 (25.0%)	*n* = 36 (30.0%)	*n* = 68 (25.7%)
No	*n* = 1242 (81.1%)	*n* = 813 (77.5%)	*n* = 257 (79.8%)	*n* = 20 (80.0%)	*n* = 9 (56.3%)	*n* = 79 (65.8%)	*n* = 173 (65.3%)
Unknown	*n* = 73 (4.8%)	*n* = 64 (6.1%)	*n* = 7 (2.2%)	*n* = 2 (8.0%)	*n* = 3 (18.8%)	*n* = 5 (4.2%)	*n* = 24 (9.1%)

*SE* superficial endometriosis, *DIE* deep infiltrating endometriosis, *AM* adenomyosis

### Pelvic pain

In 3328 patients, the chi-square test showed a significant difference in pelvic pain frequency across phenotypes (*p* < 0.001). Groups SE only, SE/AM, and SE/DIE/AM significantly contributed to the overall effect. Pairwise comparisons confirmed significant differences between multiple group pairs. Overall, pelvic pain was less frequent in group SE only and more common in groups SE/AM and SE/DIE/AM.

**Fig. 2:**
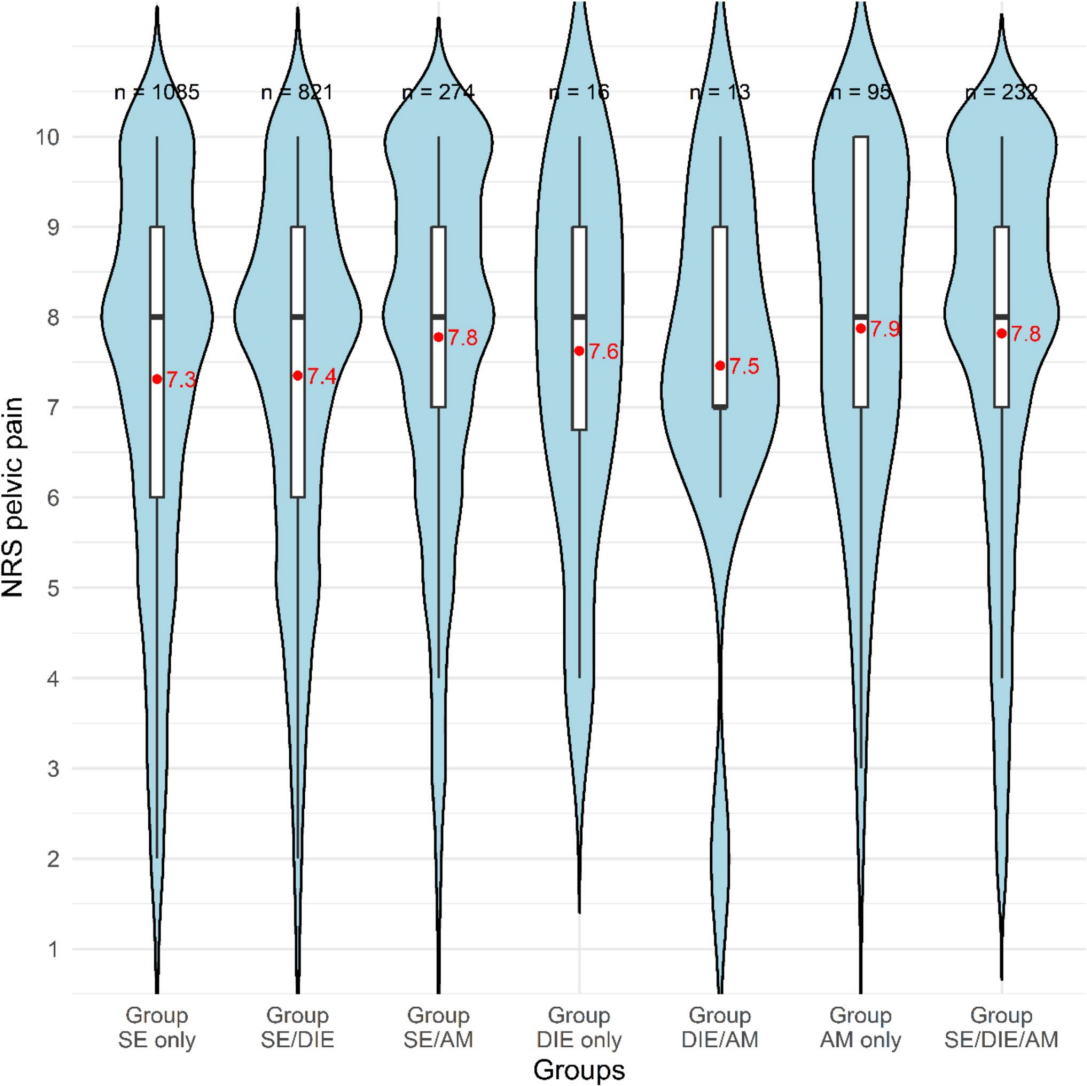
Violin plot of NRS for pelvic pain by endometriosis groups. Red dots indicate group means; black lines indicate medians

Pelvic pain (NRS) was assessed in 2536 patients. Pain scores differed significantly across groups (Kruskal–Wallis test, *p* < 0.001). Group SE only reported significantly lower pain scores compared to Group SE/AM (*p* = 0.027) and Group SE/DIE/AM (*p* = 0.006). Similarly, Group SE/DIE showed significantly lower pain scores compared to Group SE/AM (*p* = 0.032) and Group SE/DIE/AM (*p* = 0.007). The distribution of pelvic pain (NRS > 0) across the groups is shown in Fig. 2.

### Dyspareunia

Dyspareunia frequency was analyzed in 3276 patients. A significant difference was found between groups (*p* = 0.003). Groups SE only and SE/AM significantly contributed to the overall effect based on the adjusted standardized residuals. Pairwise comparison showed that dyspareunia was significantly less frequent in Group SE only and more frequent in Group SE/AM.

**Fig. 3:**
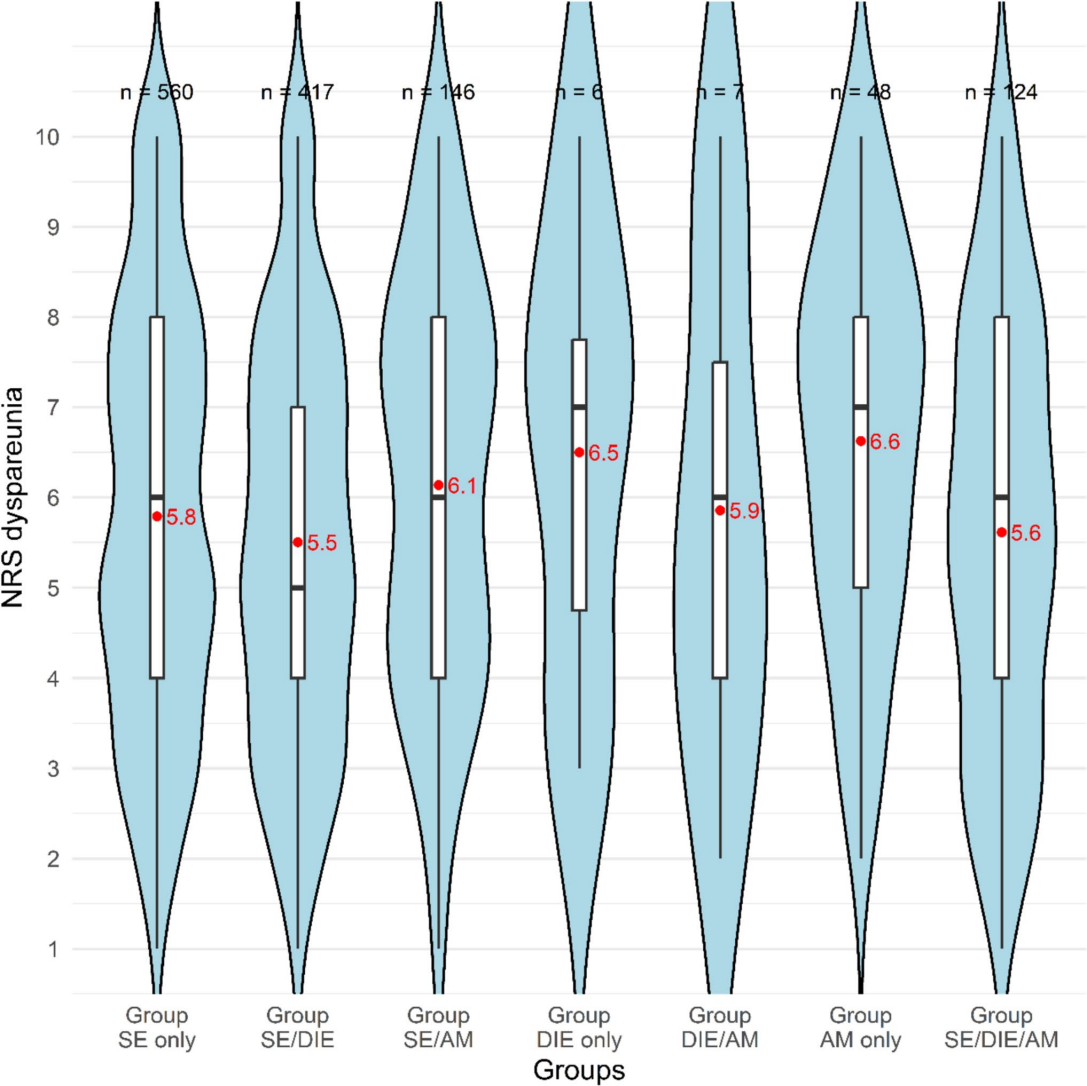
Violin plot of NRS for dyspareunia by endometriosis groups. Red dots indicate group means; black lines indicate medians

A total of 1308 patients were included in the analysis of dyspareunia, based on NRS scores to assess pain intensity. Following a statistically significant result in the Kruskal–Wallis test (*p* = 0.008), pairwise group comparisons were performed. Only Group SE/DIE showed significantly lower NRS scores for dyspareunia compared to Group AM only (*p* = 0.025). The distribution of patients reporting dyspareunia (NRS > 0) across the groups is shown in Fig. 3.

### Dysuria

Dysuria frequency was analyzed in 3312 patients. The chi-square test showed a significant difference between groups (*p* = 0.003). Group SE/DIE/AM had a significantly increased dysuria rate based on adjusted standardized residuals. In pairwise comparisons, significant differences were observed not only between group SE only and group SE/DIE/AM, but also between group SE only and group SE/AM. This finding can be explained by the fact that group SE only and group SE/AM narrowly missed the threshold for statistical significance in the residual analysis, each in opposite directions. As a result, a statistically significant difference was detected between the two groups, even though neither group SE only nor group SE/AM was individually identified as significantly deviant based on the residuals.

**Fig. 4:**
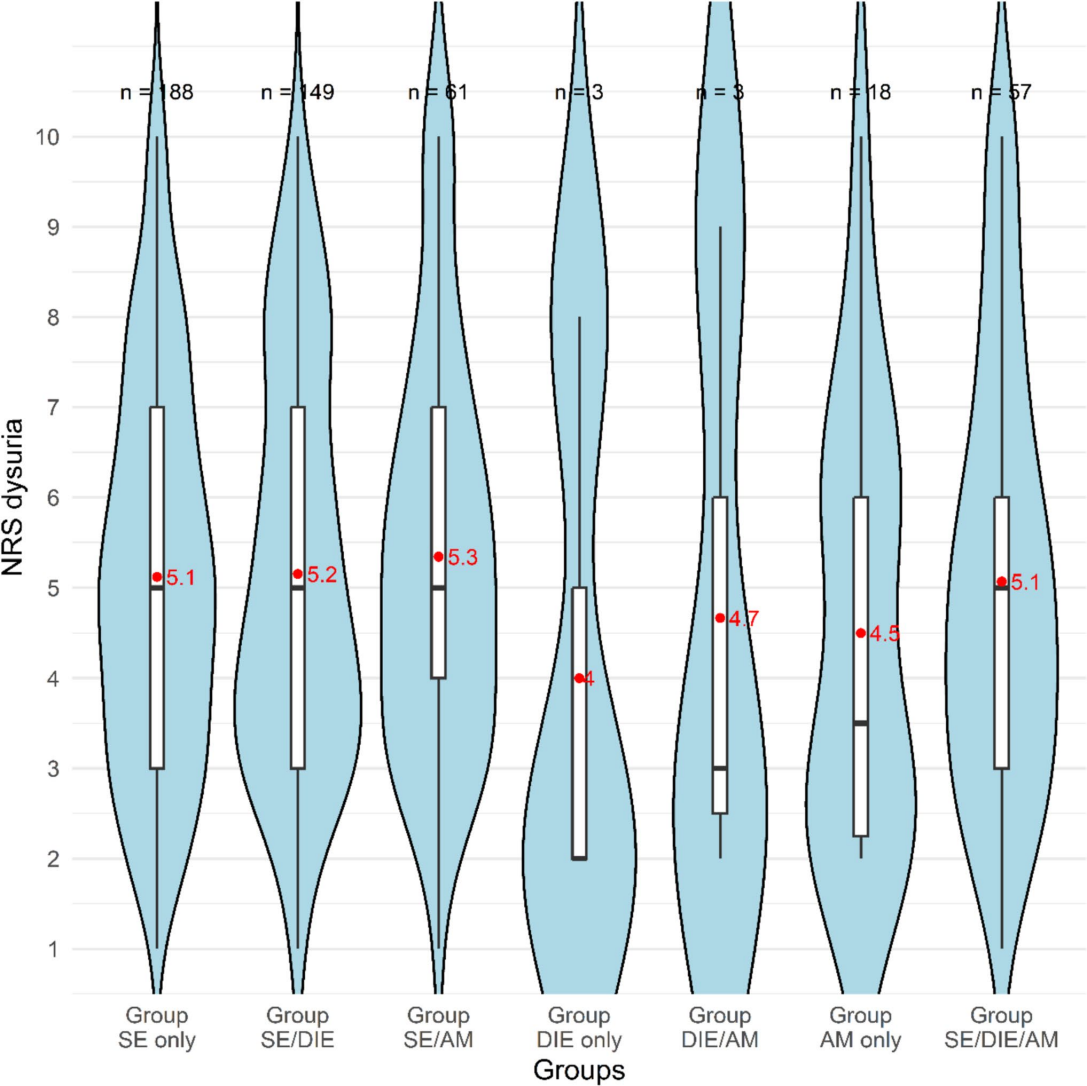
Violin plot of NRS for dysuria by endometriosis groups. Red dots indicate group means; black lines indicate medians

Pain scores for dysuria were analyzed in a cohort of 479 patients. The overall comparison between groups did not yield a statistically significant result. The distribution of patients reporting dysuria (NRS > 0) across the groups is shown in Fig. 4.

### Dyschezia

For the analysis of the pain type dyschezia, data from 3309 patients were available. The analysis yielded a statistically significant result (*p* < 0.001). Based on the standardized adjusted residuals, Groups SE only and SE/DIE/AM were identified as significantly divergent. Pairwise comparisons revealed significant differences between Groups SE only and SE/DIE, as well as between Groups SE only and SE/DIE/AM. In line with these findings, patients in Group SE only were less likely to present with dyschezia, whereas patients in Group SE/DIE/AM were more likely to report this symptom.

**Fig. 5:**
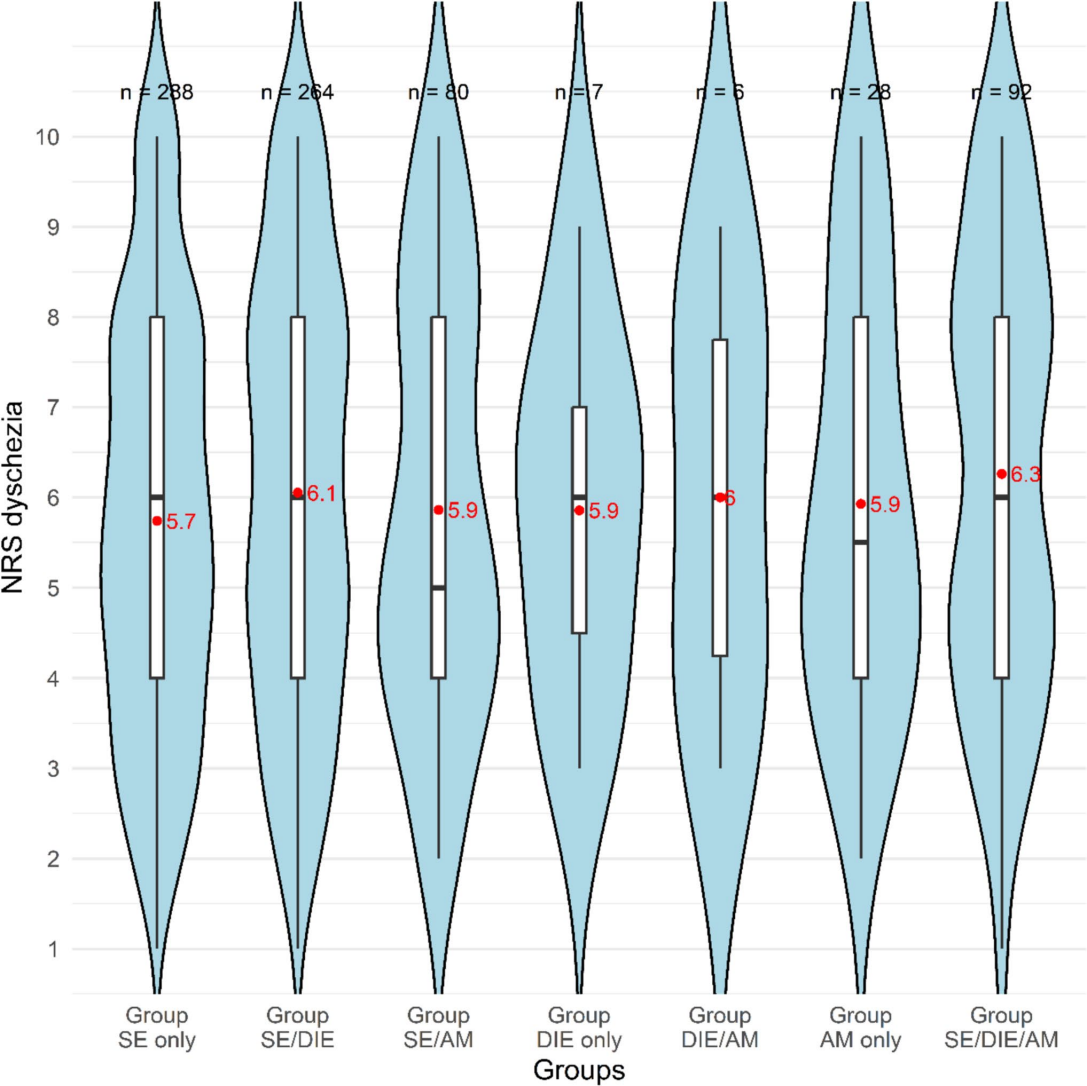
Violin plot of NRS for dyschezia by endometriosis groups. Red dots indicate group means; black lines indicate medians

The NRS for dyschezia over the past three months was assessed in a total of 765 patients. No statistically significant differences were observed between the groups. The distribution of dyschezia (NRS > 0) across the groups is shown in Fig. 5.

### Overview of frequencies

Statistically significant differences in the frequencies of pain types across endometriosis phenotype groups (based on χ^2^ tests) are presented in Supplementary Table 1.

## Discussion

In this study, seven endometriosis phenotypes were analyzed. Groups with AM presented less frequently with infertility, which contrasts with the assumption that adenomyosis is at least partly associated with impaired fertility [27]. Groups involving AM had a higher mean age, likely reflecting diagnostic bias: AM was diagnosed either sonographically or histologically via hysterectomy, typically after family planning was completed, resulting in fewer infertility-related presentations. Interestingly, while SE only presented less frequently with pain—consistent with previous reports—DIE only also showed lower pain prevalence, which contrasts with previous findings linking DIE to pain [28, 29]. However, not all studies have shown such an association, especially not for dyspareunia [30]. A higher number of live births was associated with a lower likelihood of infertility as the reason for presentation. However, variations in pregnancy and birth rates across groups could not be fully explained by age differences alone, warranting further investigation.

In our study, we demonstrated significant differences in various types of pain when patients were categorized according to their endometriosis phenotypes. These differences were most pronounced for pelvic pain. The data suggest that pelvic pain occurs less frequently in patients with SE only. Moreover, the other pain types were also reported less frequently in this group. In contrast, phenotypes involving AM were more frequently associated with pelvic pain. This finding is consistent with previous studies demonstrating a link between AM and dysmenorrhea or lower abdominal pain [31–33]. Although small subgroup sizes limited the ability to draw definitive conclusions for all phenotypes and pain types, a clear trend emerged: patients with DIE were more likely to report dyschezia. This observation aligns with existing evidence that DIE can affect the bowel, leading to altered defecation and dyschezia [29, 34]. In addition, our findings confirm previous reports suggesting an association between AM and an increased occurrence of dysuria [35]. In our study, the presence of AM in addition to SE was associated with higher rates of dyspareunia compared to SE alone. Nevertheless, this finding is based on a single significant group comparison and should be interpreted with caution. More severe dyspareunia in AM compared to those with SE has also been reported in previous studies [34, 36].

When considering only patients who presented with pelvic pain (NRS > 0), it becomes evident that those with isolated SE reported lower pelvic pain scores compared to patients from groups with an additional AM component (groups SE/AM and SE/DIE/AM). However, no significant differences were observed in comparison to the group with AM only. This finding may suggest that AM contributes to pain primarily when combined with other endometriosis subtypes, whereas isolated occurrence does not appear to be associated with increased pain intensity.

With respect to DIE, pelvic pain did not appear to be intensified by the presence of concomitant DIE. This was particularly evident when comparing Group SE/DIE with Groups SE/AM and SE/DIE/AM. No conclusions could be drawn for Group DIE only, as the sample size of 25 patients was likely too small to detect statistically significant differences.

Interestingly, among patients reporting dyspareunia (NRS > 0), those in Group SE/DIE experienced less severe pain than those in Group AM only. This finding aligns with previous data suggesting that AM plays a substantial role in the development of dyspareunia [36].

Our study has several limitations and strengths. A major limitation of the study is the diagnosis of endometriosis itself. In accordance with the current ESHRE guideline, the diagnosis of endometriosis was made intraoperatively and applied even if the endometriosis was not histologically confirmed [2]. We also opted for this procedure, as otherwise there is a high risk of overlooking patients with endometriosis. Nevertheless, histologically confirmed endometriosis was present in 2796 out of 3329 patients, corresponding to a confirmation rate of 83.99%. We diagnosed adenomyosis histologically or by a structured TVUS in a specialized ultrasound department of the Gynecology Departement of the University Hospital of Erlangen. Although two criteria are widely accepted as the cut-off for the diagnosis of AM, it is unclear whether diagnosis with more than one criterion improves the diagnosis rate [37]. We also added the “question mark sign” to the MUSA features, as this is thought to increase sensitivity and specificity [3].

A key strength of this study lies in the large number of patients and the clear grouping based on endometriosis phenotype. Another strength of this study, which is designed as a prospective cohort study, is the comprehensive and detailed data collection. Most of the data were obtained from a preoperative questionnaire and were supplemented postoperatively by clinical information extracted from the patient record. Furthermore, the use of a dedicated ultrasound unit ensured high-quality imaging and accurate identification of AM.

## Conclusion

Pain frequency and intensity varied by endometriosis phenotype, with AM showing the strongest influence. Pain was less frequent in SE only, while AM, especially in combination with other subtypes, was linked to higher rates of pelvic pain, dyspareunia, and dysuria. Among patients with pelvic pain, intensity was lowest in SE and higher when AM was present. DIE was mainly associated with dyschezia without increasing pelvic pain intensity.

Our findings highlight the need to consider both anatomical phenotype and clinical parameters in endometriosis management. Integrating phenotype, symptom patterns, fertility status, and comorbidities could improve disease understanding, enable individualized treatment, and enhance prognostic accuracy. This multidimensional approach should be further validated in future research and clinical guidelines.

## Supplementary Information

Below is the link to the electronic supplementary material.

Supplementary file1 (DOCX 15 KB)

## Data Availability

No datasets were generated or analysed during the current study.
