# Supplementary material for: Clinical characterization of endometriosis phenotypes
Source: Arch Gynecol Obstet. 2025 Oct 17;312(6):2089–100. doi: 10.1007/s00404-025-08191-4 (PMC12705834; doi:10.1007/s00404-025-08191-4)
Supplement: Supplementary file 1 — Supplementary file1 (DOCX 15 KB) [file 404_2025_8191_MOESM1_ESM.docx]

**Supplementary tables**

**Supplementary table 1.** Statistically significant differences in the frequencies of pain types across endometriosis phenotype groups (based on Chi² tests).

|  | Group SE only | | | | Group SE/DIE | | | | Group SE/AM | | | | Group DIE only | | | | Group SE/DIE/AM | | | |
| --- | --- | --- | --- | --- | --- | --- | --- | --- | --- | --- | --- | --- | --- | --- | --- | --- | --- | --- | --- | --- |
|  | **PP** | **DP** | **DU** | **DC** | **PP** | **DP** | **DU** | **DC** | **PP** | **DP** | **DU** | **DC** | **PP** | **DP** | **DU** | **DC** | **PP** | **DP** | **DU** | **DC** |
| **Group SE only** |  | | | |  |  |  |  |  |  |  |  |  |  |  |  |  |  |  |  |
| **Group SE/DIE** |  |  |  |  |  | | | |  |  |  |  |  |  |  |  |  |  |  |  |
| **Group SE/AM** |  |  |  |  |  |  |  |  |  | | | |  |  |  |  |  |  |  |  |
| **Group DIE only** |  |  |  |  |  |  |  |  |  |  |  |  |  | | | |  |  |  |  |
| **Group SE/DIE/AM** |  |  |  |  |  |  |  |  |  |  |  |  |  |  |  |  |  | | | |

Colors are interpreted row-wise. Light gray = less frequent pain; dark gray = more frequent pain.

SE: superficial endometriosis, DIE deep infiltrating endometriosis, AM: adenomyosis, PP: pelvic pain, DP: dyspareunia, DU dysuria, DC dyschezia
